# Predictors of mortality in hospitalized COVID-19 patients: A Mexican population-based cohort study

**DOI:** 10.37796/2211-8039.1124

**Published:** 2021-06-01

**Authors:** Fernando Mesta, Angel M. Coll, Miguel Á. Ramírez, Livan Delgado-Roche

**Affiliations:** a Department of Occupational Health, Safety and Hygiene Research, National Medicine and Homeopathy School of the National Polytechnic Institute, 239 Guillermo Massieu Helguera Ave, La Escalera, 07320, Mexico City, Mexico; b AbbVie Farmacéuticos, S.A. de C.V. 3720 Building 3, Office 10-001, Jardines Del Pedregal, 01900, Mexico City, Mexico; c Laboratorios Liomont, S.A. de C.V. 5420-12th Floor, México-Toluca Rd, El Yaqui, Cuajimalpa, 05320, Mexico City, Mexico

**Keywords:** COVID-19, SARS-CoV-2, Tracheal intubation, Risk factors, Death

## Abstract

**Objectives:**

COVID-19 outbreak brings a challenge to healthcare systems. The sex, age, and cardiometabolic comorbidities have been considered risk factors for disease severity. To evaluate the association between risk factors with death as well the risk of death in hospitalized COVID-19 patients.

**Methods:**

The present cross-sectional cohort study, includes hospitalized SARS-CoV-2 confirmed cases. Data analysis was performed using the National COVID-19 Cases Report Database. Pearson’s χ^2^ test and odds ratios (95% CI) were calculated to determine the association between variables. Thereafter, risk of death was evaluated by Cox proportional hazards model.

**Results:**

A total of 67 328 inpatients were included; mean age 55.29 years (±15.97). Of total, 42 164 (62.62%) were men, 6 349 (9.43%) were intubated, and 23 873 (35.46%) died. Male sex, age older than 60 years, and cardiometabolic comorbidities were associated with death. Hazard ratio for death in older intubated patients was lower than in non-intubated (HR 1.242, 95%CI, 1.167–1.322; *P* < 0.001) and (HR 2.128, 95%CI, 2.066–2.193; *P* < 0.001) respectively.

**Conclusions:**

Tracheal intubation or not is the most important predictor for death in COVID-19 infected patients in this Mexican cohort. Already known risk factors for COVID-19 severity may become less relevant once patients require tracheal intubation.

## 1. Introduction

COVID-19, caused by severe acute respiratory syndrome coronavirus 2 (SARS-CoV-2), shows a global case fatality rate of 4.9%, although in countries like Mexico or Italy has been reported as high as 12.3% and 14.45%, respectively. At present (July 13th, 2020), COVID-19 has affected 13 070 590 people and has caused 572 428 deaths [1]. Therefore, the ongoing COVID-19 outbreak brings a big challenge to the hospital services, in particular intensive care units (ICU). In order to optimize the availability of ICU resources could be imperative to identify patients at high risk of progressing to severe and critical stages [2]. Tracheal intubation in COVID-19 patients seems to be a risk to physiologically compromised patients [3,4]. In addition, comorbidities including diabetes, obesity, and hypertension have been associated with poor clinical outcomes [5]. An older age, diabetes and history of cardiovascular disease, especially hypertension, but also chronic heart failure and coronary artery disease among others, are between the most important risk factors for COVID-19 mortality [6–8]. In Mexico, as in other developing countries with a high burden of these cardiometabolic comorbidities, the scenario seems to be aggravated [1,5,6]. Thus, we aimed to evaluate if age, sex, and preexisting comorbidities are risk factors for death in a Mexican cohort of hospitalized patients, as well as in intubated patients with COVID-19.

## 2. Methods

The present is a cross-sectional cohort study, including adult (>18 years) hospitalized SARS-CoV-2 confirmed cases of all ages from February 28th to June 29th, 2020. The infection was confirmed by reverse transcription polymerase chain reaction (RT-PCR), performed according to the protocol approved by the Institute of Epidemiological Diagnosis and Reference [9]. Data analysis was carried out using the National COVID-19 Cases Report Database (Mexican Department of Epidemiology) which is of public access [9,10]. Foreign subjects were excluded from the analyses, and incomplete data from the database were eliminated.

### 2.1. Statistical Analysis

The statistical analysis was performed by using the SPSS 24.0 software (IBM, USA). Pearson’s χ^2^ test and odds ratios (OR) with 95% confidence intervals (CI) were calculated to determine the association between age, sex, tracheal intubation, and comorbidities (diabetes, hypertension and obesity) with death in hospitalized patients. Age was considered as a categorical variable (<60, ≥60 years old) in all the analyses. Thereafter, the risk of death was evaluated by Cox proportional hazards model in intubated patients in comparison with non-intubated patients. Values of p < 0.05 were considered statistically significant.

## 3. Results

Until June 29^th^, 220 657 SARS-CoV-2 accumulated confirmed cases were registered in Mexico. Of total, 68 296 (30.95%) patients have required hospital care. Of total inpatient SARS-CoV-2 confirmed cases, 161 foreigners (0.24%) were excluded; and 807 cases (1.18%) were eliminated because of incomplete data. Thus, a total of 67 328 COVID-19 inpatients were included in the analyses. The mean age was 55.29 years (±15.97), 42 164 (62.62%) were men, and the total number of deaths was 23 873 (35.46%). Of hospitalized patients, 6 349 (9.43%) required tracheal intubation. The mean age of intubated patients was 57.16 years (±15.25), 4 292 (67.60%) were males, and 4 280 (67.41%) died.

The present analysis showed that sex, age, hypertension, diabetes, obesity, and tracheal intubation were significantly associated with death in COVID-19 hospitalized patients (Table 1).

**Fig. 1:**
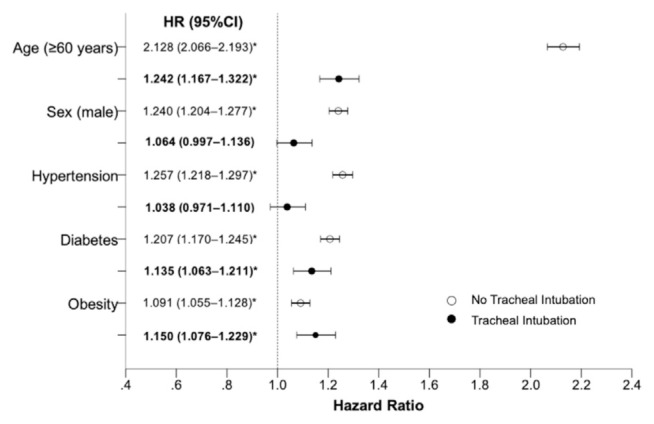
Hazard ratio for death according to risk factors in hospitalized SARS-CoV-2 patients, according to intubation requirement. HR, hazard ratio. *P < 0.001.

Survival analysis of hospitalized patients that didn’t require tracheal intubation showed that men with SARS-CoV-2 infection had a higher risk of death in comparison with women (HR 1.240, 95%CI, 1.204 to 1.277; *P* < 0.001). Age older than 60 years was associated with more than double the risk of mortality (HR 2.128, 95%CI, 2.066 to 2.193; *P* < 0.001). In addition, hypertension (HR 1.257, 95%CI, 1.218 to 1.297; *P* < 0.001), diabetes (HR 1.207, 95%CI, 1.170 to 1.245; *P* < 0.001), and obesity (HR 1.091, 95%CI, 1.055 to 1.128; *P* < 0.001), significantly increased the risk of death in non-intubated hospitalized patients (Fig. 1). In contrast, the sex and hypertension were not associated with an increased risk of death in intubated patients; (HR 1.064, 95%CI, 0.997 to 1.110; *P* = 0.062) and (HR 1.038, 95%CI, 0.971 to 1.110; *P* = 0.268), respectively. Intubated patients with age older than 60 years, diabetes or obesity had a discrete increased risk of death; (HR 1.242, 95%CI, 1.167 to 1.322; *P* < 0.001), (HR 1.135, 95%CI, 1.063 to 1.211; *P* < 0.001), and (HR 1.150, 95%CI, 1.076 to 1.229; *P* < 0.001) (Fig. 1).

**Fig. 2:**
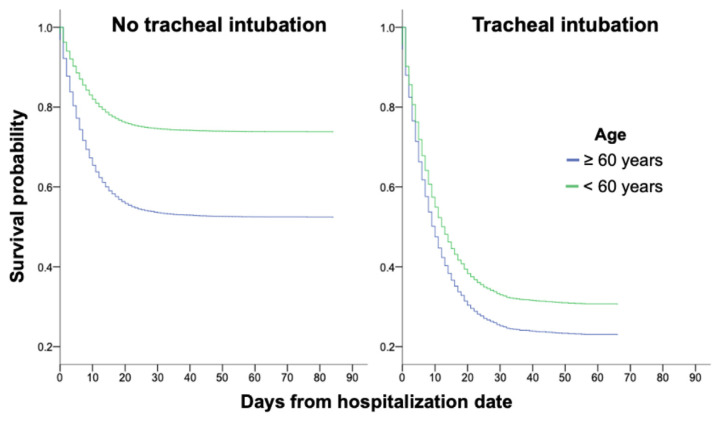
Survival probability of patients with COVID-19, according to days from hospitalization date, age and intubation requirement.

In addition, the survival analysis showed that the age had a different impact on survival probability between non-intubated and intubated population. In intubated patients, survival probability was drastically diminished regardless of age (Fig. 2).

## 4. Discussion

Retrospective observational studies have shown that patients with COVID-19 who are older, male, or diabetic are at higher risk of requiring intubation [11,12]. The association was also pointed out in a cross-sectional cohort study performed in Mexican population, identifying obesity as a risk factor for mechanical ventilation [11]. In the same way, these risk factors, including hypertension have been associated with death in SARS-CoV-2 infected patients [13–15]. However, available data may not be enough to mark safe conclusions on the prognosis of COVID-19 patients who require mechanical ventilation [16].

The present results are in accordance with previous studies that recognize older age, male sex, hypertension, diabetes and obesity as risk factors for death and severity in SARS-CoV-2 patients [17–20]. In addition, here we showed evidence of association between the requirements of tracheal intubation with death in COVID-19 patients. However, the sex and hypertension seem to be clinically irrelevant regarding the risk of death once the patient is intubated. Likewise, the age of intubated patients didn’t appear to be as relevant as in non-intubated population. This finding suggests that younger age is not a protective factor in intubated patients as occurs in hospitalized but not intubated population. Obesity, unlike the other evaluated risk factors, showed a higher HR in intubated patients. This may be explained in part by the negative effects of obesity in mechanics of the lungs and airway [21]. In fact, COVID-19 severity has been correlated with higher body mass index [22].

Although the limitations of this cross-sectional cohort study, it may contribute to understand how age, sex, and cardiometabolic risk factors influence COVID-19 outcome in critically ill patients. Of note, some of these risk factors for COVID-19 may become clinically less relevant once patients deteriorate and require TI. Therefore, SARS-CoV-2 infection needs to be widely understood and further evidences regarding the pathophysiology and risk factors are still required.

## Figures and Tables

**Table 1:** Association of age, sex, comorbidities and intubation with death.

Total population: 67 328	Death, n (%)		χ2 test		
		
	Yes	No			
			
	23 873 (35.5)	43 455 (64.5)	OR (95% CI)	RR (95% CI)	P value
Age, ≥ 60 years, n (%)	13 528 (19.7)	14 293 (21.1)	2.563 (2.481–2.647)	1.809 (1.773–1.846)	<0.001
Sex, male, n (%)	15 676 (23.3)	26 488 (39.3)	1.225 (1.185–1.266)	1.141 (1.117–1.166)	<0.001
Hypertension, n (%)	10 140 (15.1)	13 011 (19.3)	1.728 (1.672–1.785)	1.409 (1.381–1.438)	<0.001
Diabetes, n (%)	8 893 (13.2)	11 867 (17.6)	1.580 (1.528–1.634)	1.332 (1.305–1.359)	<0.001
Obesity, n (%)	5 910 (8.8)	9 981 (14.8)	1.103 (1.063–1.145)	1.065 (1.040–1.090)	<0.001
Tracheal intubation, n (%)	4 280 (6.4)	2 069 (3.1)	4.370 (4.135–4.617)	2.098 (2.055–2.142)	<0.001

COVID-19 hospitalized patients with age older than 60 years, male sex, comorbidities history (hypertension, diabetes and obesity) or requiring tracheal intubation were more likely to death than SARS-CoV-2 hospitalized patients without those risk factors. Total population of SARS-CoV-2 confirmed patients hospitalized in Mexico from February 28th to June 29th, 2020, according to National COVID-19 Cases Report Database (Mexican Department of Epidemiology).
